# Incidence of Cervical Kyphosis and Factors Associated with Improvement in Postoperative Cervical Spinal Alignment in Idiopathic Scoliosis with Major Thoracolumbar/Lumbar and Thoracic Curves

**DOI:** 10.3390/jcm13133811

**Published:** 2024-06-28

**Authors:** Kai Mizukami, Tetsuro Ohba, Nobuki Tanaka, Kotaro Oda, Marina Katsu, Hayato Takei, Goto Go, Hirotaka Haro

**Affiliations:** Department of Orthopaedic Surgery, University of Yamanashi, Yamanashi 409-3898, Japan; kmizukami@yamanashi.ac.jp (K.M.); tanakan@yamanashi.ac.jp (N.T.); koda@yamanashi.ac.jp (K.O.); marinak@yamanashi.ac.jp (M.K.); takeih@yamanashi.ac.jp (H.T.); goto.gtg97@gmail.com (G.G.); haro@yamanashi.ac.jp (H.H.)

**Keywords:** adolescent idiopathic scoliosis, cervical kyphosis, posterior spinal fusion, thoracic kyphosis

## Abstract

**Background:** This study aimed to compare the incidence and severity of cervical kyphosis before and after surgery between patients with adolescent idiopathic scoliosis (AIS) with major thoracolumbar/lumbar curves (Lenke type 5C group) and those with major thoracic curves (Lenke type 1A group). Further, factors associated with cervical spinal alignment changes after surgery in the two groups were examined. **Methods:** This study included consecutive patients with AIS who underwent posterior spinal fusion for Lenke type 1A and 5C curves and who were followed up for at least 1 year. To measure changes in sagittal alignment, all patients underwent radiography before, immediately after, and at 1 year after surgery. The correlation coefficients change the value of the C2–C7 angle before and after surgery (ΔC2–ΔC7) and other spinopelvic parameters were examined. **Results:** In total, 19 of 30 patients in the Lenke type 1A group and 21 of 36 in the Lenke type 5C group presented with cervical kyphosis preoperatively. Hence, the incidence of cervical kyphosis did not significantly differ between the two groups. Further, the two groups had significantly higher thoracic kyphosis (TK) and greater C2–C7 angles postoperatively. The TK of the Lenke type 5C group further increased at 1 year postoperatively. The Lenke 1A type group presented with a significant re-decrease in the C2–C7 angle at 1 year postoperatively. However, the C2–C7 angle of the Lenke type 5C group did not change. The ΔTK was closely associated with the ΔC2–ΔC7 in the Lenke type 1A group, but not in the Lenke type 5C group. **Conclusions:** In thoracic AIS, postoperative cervical alignment should achieve an adequate TK and promote correction of the coronal plane curve. Moreover, selective corrective surgery can improve postoperative cervical alignment in lumbar AIS.

## 1. Background

Adolescent idiopathic scoliosis (AIS) is a relatively common disease presenting as a three-dimensional spinal deformity. Approximately 2–3% of the general population presents with AIS. Coronal alignment has traditionally been the focus of AIS [1]. However, recent studies have found that sagittal alignment is also important. The Lenke classification is widely used for categorizing an AIS based on coronal plane deformities [2]. The Lenke classification is highly valued for its reproducibility and reliability in both clinical and research settings. It facilitates standardized communication among healthcare professionals and guides treatment decisions based on specific curve patterns and modifiers [3]. However, recent studies indicate that it may not adequately address sagittal plane alignment, which is crucial for a comprehensive understanding of spinal deformities. Research emphasizes the importance of evaluating sagittal alignment and global spinal balance in AIS, suggesting that additional parameters or new classification systems might be necessary to better capture sagittal plane deformities in these patients [4,5]. Although still controversial, sagittal cervical alignment was found to affect the health-related quality of life of patients with AIS [6,7,8,9,10,11]. Further, patients with AIS have a higher incidence of reduced thoracic kyphosis (TK) and cervical lordosis than healthy individuals [2,3,4,5,6]. The biomechanical foundations of scoliosis treatment substantiate the validity of therapeutic goals in several critical ways. Initially, scoliosis treatment typically begins with bracing, which aims to prevent curve progression and maintain trunk stability. Bracing works by applying strategic passive forces to correct spinal curvature while also encouraging active muscle contraction, thus promoting a more balanced posture. However, in cases where scoliosis progresses despite conservative measures, surgical intervention becomes necessary. Surgical treatments, such as spinal fusion and instrumentation, are designed to correct the deformity, halt further progression, and restore proper alignment in both the sagittal and coronal planes. This dual approach ensures comprehensive management of scoliosis, addressing both immediate and long-term biomechanical challenges associated with the condition. Changes in sagittal alignment after spinal corrective surgery have been gaining attention. Selective correction surgery of the thoracic spine must achieve an adequate TK to prevent flat back syndrome and the consequent loss of lumbar lordosis (LL) [7]. In AIS with thoracic curves, reciprocal changes in the cervical spine occur due to TK changes after corrective spinal fusion [8,9,10]. In contrast, the effects of selective correction for AIS of the thoracolumbar (TL) curves on the sagittal alignment of the thoracic and cervical spine are still not completely elucidated. The pathology of cervical kyphosis before and after surgery in AIS is still unknown. Therefore, the differences between thoracic and lumbar curves should be examined. We hypothesized that there may be variations in the frequency and severity of cervical kyphosis in AIS based on whether the primary curve is thoracic or lumbar. Additionally, we anticipated differences in the changes in cervical alignment preoperatively and postoperatively.

This study aimed to compare the incidence and severity of cervical kyphosis before and after surgery between patients with AIS with major thoracolumbar/lumbar curves (the Lenke type 5C group) and those with AIS with major thoracic curves (the Lenke type 1A group). Further, factors associated with cervical spinal alignment changes after surgery in the two groups were examined.

## 2. Methods

### 2.1. Ethics Approval

The institutional review board approved this study. Written informed consent was obtained from the parents or guardians of all participants. The criteria for surgery were primarily a lumbar curve greater than 40 degrees and a thoracic curve greater than 45 degrees. However, the final decision was made with the agreement of the primary physician and the patient’s parents. This study included patients who underwent posterior corrective fusion surgery for AIS with Lenke 1A or Lenke 5C at our institution and were followed up for one year postoperatively.

### 2.2. Baseline Characteristics of Patients with AIS

The medical records of 66 consecutive patients with AIS were reviewed retrospectively. This study included eligible patients with AIS with major thoracolumbar/lumbar curves (Lenke type 5C) or main thoracic curves (Lenke type 1A) who underwent posterior spinal fusion (PSF) surgery between July 2018 and August 2022 and who were followed up for at least 24 months at our university hospitals.

This study included 30 consecutive patients with AIS (3 men and 27 women) who underwent PSF for Lenke type 1A curves, and 36 consecutive patients with AIS (1 men and 35 women) who underwent PSF for Lenke type 5C curves. The two groups significantly differed in terms of age, sex, height, weight, and body mass index. Table 1 presents the number of fixed vertebrae and the spinal level of the upper instrumented vertebrae and lower instrumented vertebrae.

### 2.3. Surgical Procedure

All patients were positioned prone on a Jackson table (Mizuho OSI, Union City, CA, USA). The inferior articular process within the fixed range was removed before the insertion of pedicle screws (PSs) in every case, with no additional osteotomies performed either before or after the PS fixation. A spinous process bone clamp, attached to a reference frame, was positioned at the uppermost level visible in the O-arm field of view. To reduce the number of O-arm images needed, as many screws as possible were inserted within one field of view. The screws were inserted from the left side first when viewed posteriorly. The O-arm system (Medtronic, Louisville, CO, USA) was used to capture intraoperative images, which were then sent to the Stealth computer navigation system (Medtronic Navigation, Louisville, CO, USA). The navigated instruments were subsequently registered. A digitally guided awl was used to enter the center of the pedicle canal, guided by image data in the axial, coronal, and sagittal planes. Taps and pedicle screws were placed using these navigated instruments.

### 2.4. Radiographic Parameters

**Figure 1:**
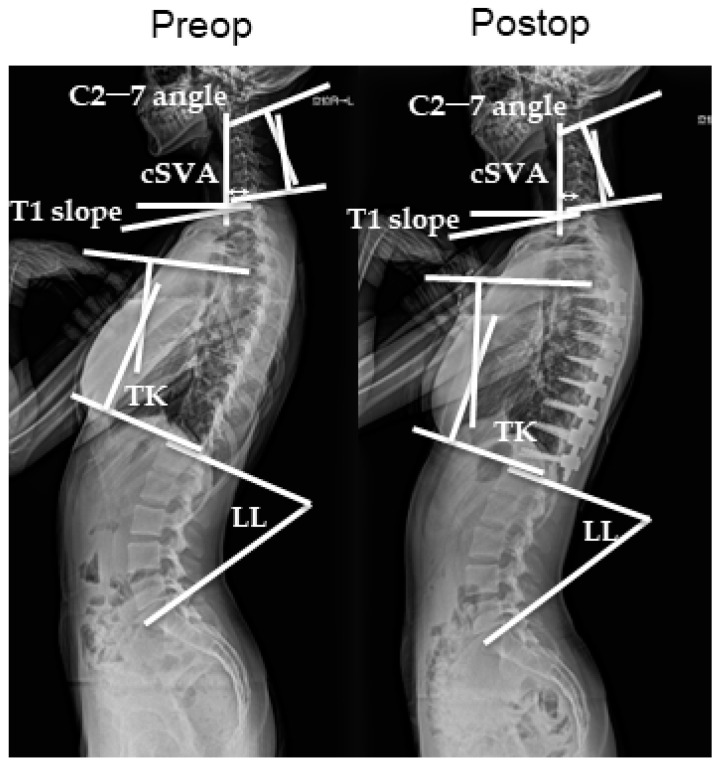
Standing lateral X-ray showing the calculation scheme before and after surgery.

All patients underwent radiological evaluation of the whole spine before, immediately after, and at 1 year after surgery. Anterior and lateral radiographic images of the whole spine were obtained in the standing position. On the lateral view, the patients stood with the knees locked, feet shoulder-width apart, elbows bent, and finger joints in the supraclavicular fossa on each side and looked straight ahead. The Cobb angles were measured using the previously mentioned whole-spine radiographic images. The major curves were measured with the Lenke classification system. Using the same images, the sagittal alignment of the cervical spine was assessed using the following parameters: sagittal C2–C7 angles, segmental angles, and C2–7 SVA (sagittal vertical axis), which is the distance between the center of the C2 vertebral body and the posterosuperior corner of the C7 upper-end plate. Global cervical curvature (C2–C7 angle) and segmental angles were measured using the Harrison posterior tangent method. The sagittal C2–7 angle is measured with positive values indicating lordosis and negative values indicating kyphosis. T5–12 thoracic kyphosis (TK) and lumbar lordosis (LL) were also measured (Figure 1). All data were expressed as mean ± standard error of three independent measurements. Categorical variables were expressed as percentages. Radiographic measurements were obtained by two board-certified spinal surgeons (KM and TO).

### 2.5. Statistical Analysis

Mean ± SD values were reported for continuous variables or number (percentage) values were used for categorical variables. We performed Student’s *t*-tests, the Mann–Whitney test, or Fisher’s exact test to compare the mean values between the two groups. We used the Kruskal–Wallis test for statistical analysis to compare the three groups preoperatively, postoperatively, and one year after surgery. The validity of the parameters determined on sagittal or coronal radiography were individually compared using the Pearson correlation coefficients. A correlation coefficient of 0.00–0.25 indicated a minimal relationship; 0.25–0.50, a fair relationship; 0.50–0.75, a moderate to good relationship; and >0.75, a good to excellent relationship. The asterisks indicated statistical significance (*p* < 0.05). Statistical values were calculated using Prism (version 9.0; GraphPad Software, La Jolla, CA, USA).

## 3. Results

### 3.1. Radiographic Evaluations

**Figure 2:**
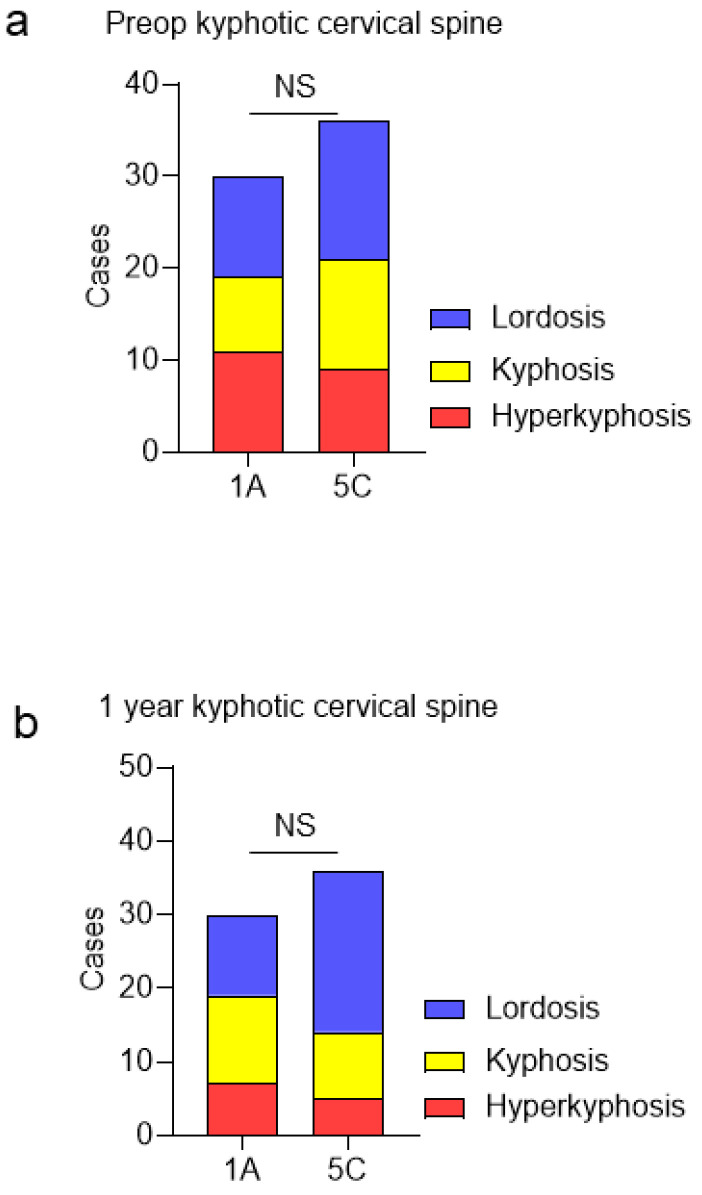
(**a**) Incidence of preoperative cervical kyphosis between Lenke type 1A and 5C groups. (**b**) Incidence of cervical kyphosis at 1 year postoperatively between Lenke type 1A and 5C groups. NS, No significant difference.

The main thoracic curve of the Lenke type 1A group was significantly corrected from 46.4° ± 11.2° preoperatively to 13.1° ± 7° postoperatively. The thoracolumbar/lumbar curve of the Lenke type 5C group was significantly corrected from 37.2° ± 9.6° preoperatively to 14.0° ± 6.7° postoperatively. Before surgery, 11, 8, and 11 patients in the Lenke type 1A group presented with cervical lordosis (C2–7 angle ≥ 0°), cervical kyphosis (C2–7 angle < 0°), and cervical hyperkyphosis (C2–7 angle < −10°), respectively (Figure 2a). Further, 15, 12, and 9 patients in the Lenke type 5C group presented with cervical lordosis, cervical kyphosis, and cervical hyperkyphosis, respectively, before surgery. The two groups did not significantly differ in terms of the incidence of cervical kyphosis and hyperkyphosis preoperatively. Postoperatively, 11, 7, and 12 patients in the Lenke type 1A group presented with cervical lordosis (C2–7 angle ≥ 0°), cervical kyphosis (C2–7 angle < 0°), and cervical hyperkyphosis (C2–7 angle < −10°), respectively (Figure 2b). Moreover, 22, 9, and 5 patients in the Lenke type 5C group presented with cervical lordosis, cervical kyphosis, and cervical hyperkyphosis, respectively. The two groups did not significantly differ in terms of the incidence of cervical kyphosis and hyperkyphosis.

**Figure 3:**
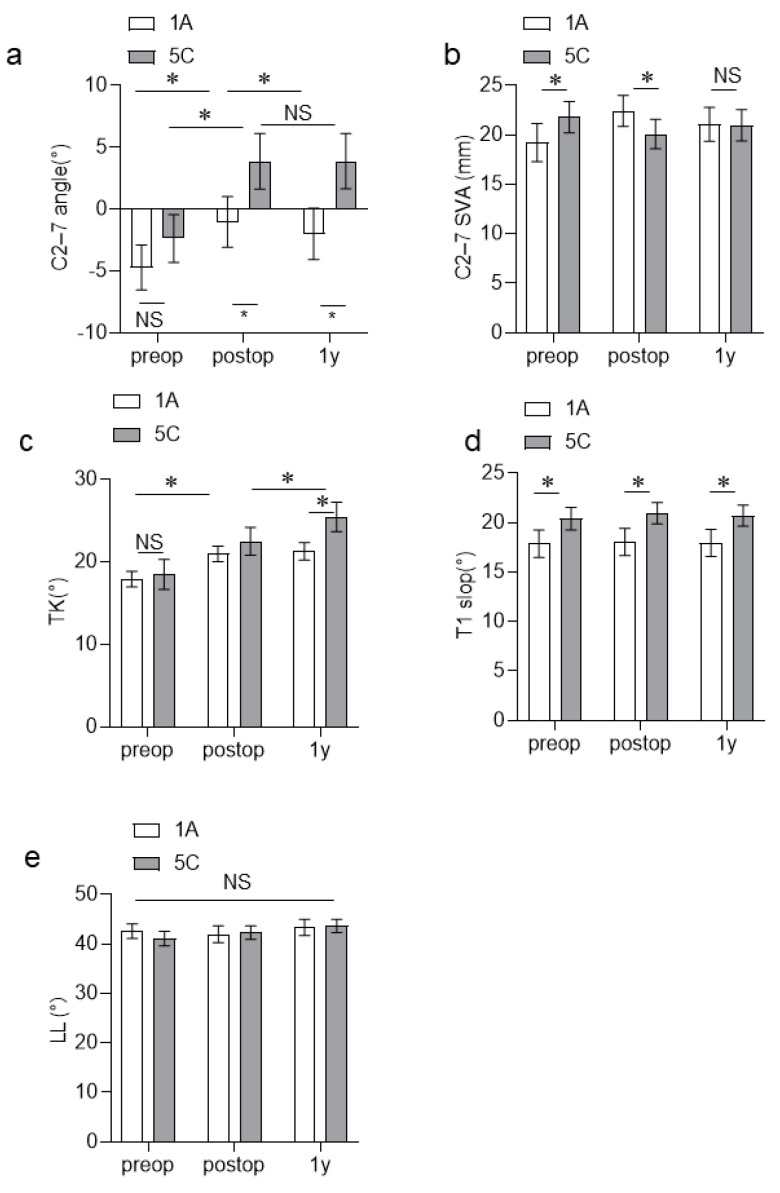
(**a**–**e**) Changes in the spinal parameters between the Lenke type 1A and 5C groups before, immediately after, and at 1 year after surgery. NS, No significant difference; * *p* < 0.05.

The C2–C7 angle of the Lenke type 1A group increased immediately after surgery but decreased significantly at 1 year after surgery. In contrast, the C2–C7 angle of the Lenke type 5C group increased significantly immediately after surgery, and it did not change at 1 year postoperatively (Figure 3a). The C2–C7 angle after surgery did not significantly differ between the two groups. However, the Lenke type C5 group had a significantly larger C2–C7 angle than the Lenke type 1A group at 1 year postoperatively (Table 2, Figure 3a). The C2–C7 SVA of the two groups did not change significantly before, immediately after, and at 1 year after surgery. The TK of the Lenke type 1A group significantly increased immediately after surgery, and it did not change at 1 year postoperatively. The TK of the Lenke type 5C group significantly increased immediately after surgery and further increased at 1 year after surgery. The two groups did not significantly differ in terms of pre- and postoperative TK. At 1 year postoperatively, the Lenke type 5C group had a significantly larger TK than the Lenke type 1A group (Table 2, Figure 3c). The Lenke type 5C group had a significantly higher T1 slope than the Lenke type 1A group before, immediately after, and at 1 year after surgery (Table 2, Figure 3d). The two groups did not present with changes in the C2WC7 SVA and LL before, immediately after, and at1 year after surgery (Figure 3b,e).

### 3.2. Association between C2–C7 Angle and Other Radiographic Parameters

**Figure 4:**
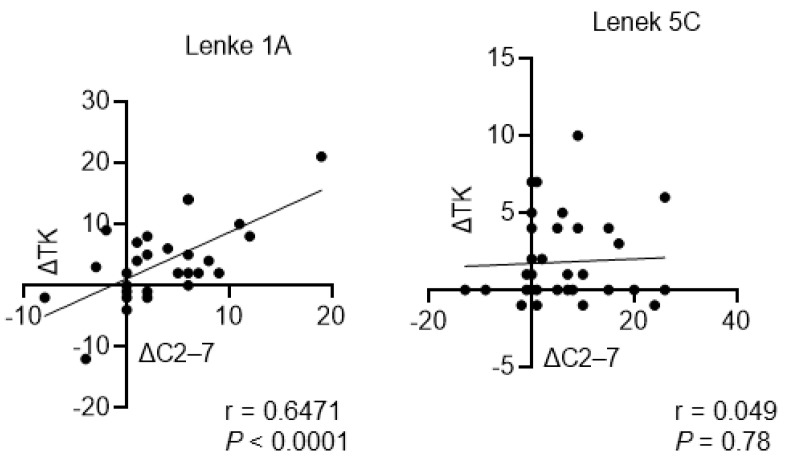
Association between ΔC2–ΔC7 angle and ΔTK in Lenke type 1A and 5C groups ΔTK, thoracic kyphosis before and after surgery; ΔC2WeΔC7 angle, C2–C7 angle before and after surgery.

Table 3 and Table 4 show the associations between the C2–C7 angle and other radiographic parameters. In the Lenke type 1A group, the C2–C7 angle was significantly associated with the T1 slope and TK before surgery. In addition, there was a stronger positive association between the C2–C7 angle and T1-slop and TK at 1 year postoperatively (Table 3). However, the other parameters were not associated with the C2–C7 angle. In the Lenke type 5C group, only the T1 slope was significantly associated with the C2–C7 angle before and after surgery. Moreover, there was no significant association between the C2–C7 angle and other parameters including TK (Table 4). Interestingly, the C2–C7 angle changes before and after surgery (ΔC2–ΔC7) were significantly associated with TK changes before and after surgery (ΔTK) in the Lenke type 1A group, but not in the Lenke type 5C group (Figure 4).

## 4. Discussion

In the current study, 19 (63%) of 30 patients in the Lenke type 1A group and 21 (58%) of 36 in the Lenke type 5C group presented with cervical kyphosis preoperatively. Hence, the incidence of cervical kyphosis did not significantly differ between the two groups. In previous reports, the prevalence of cervical kyphosis in patients with AIS is approximately 50–60%, which is significantly higher compared to 35% in control groups [12,13]. Similar to previous reports, our study showed that patients with AIS had a high prevalence of cervical kyphosis [12,13,14,15,16,17,18,19,20]. The development of cervical kyphosis among youths is still controversial. That is, some reports showed that it affects health-related quality of life, and others have contrasting results [6,7,8,9,10,11,20]. However, the pathophysiology of cervical kyphosis in AIS should be identified as it is likely to cause issues as the patient ages [6,9]. Previous studies have shown that cervical kyphosis is associated with a decreased TK in AIS. However, in the current study, the incidence of cervical kyphosis was similar between patients with AIS with thoracic curves and those with AIS with lumbar curves. Interestingly, the preoperative TK of AIS with lumbar curve and thoracic curve decreased. However, the results did not significantly differ, thereby indicating a pathology other than TK based on the presence of a structural curve.

Further, this study showed that the Lenke type 1A and 5C groups presented with a significant increase in TK and C2–C7 angles postoperatively. Interestingly, the TK of the Lenke type 5C group further increased at 1 year postoperatively. The Lenke type 1A group presented with a significant re-decrease in the C2–C7 angle at 1 year postoperatively. However, the C2–C7 angle of the Lenke type 5C group did not change (Figure 3a,c). Similar to previous reports, this study revealed that the ΔTK was closely associated with the ΔC2–C7 angle in the Lenke type 1A group [21,22], but not in the Lenke type 5C group. In AIS with thoracic curves, the changes in the C2–C7 angle after surgery are considered compensatory changes associated with surgically induced TK changes [23,24,25,26,27]. However, our study showed that different mechanisms might be associated with postoperative cervical alignment changes in AIS with lumbar curves. To the best of our knowledge, this study first compared the cervical alignment of AIS with the thoracic curve and AIS with the lumbar curve. In addition, it first revealed that the Lenke type 5C group had a significantly higher T1 slope than the Lenke type 1A group. However, the TK values did not significantly differ (Figure 3c,d). Based on previous studies, Lenke type 5C and IA curves were closely associated with the C2–C7 angle and T1 slope before and after surgery [21,22]. There were no significant changes in the LL before and after surgery (Figure 4). However, the TK of the Lenke type 5C group increased significantly from the immediate postoperative period to 1 year postoperatively. Therefore, there might have been changes in not only the degree of TK and LL but also the harmony of the spinal curvature. Recently, there has been increasing attention on cervical sagittal alignment in patients with AIS. It has been suggested that not only the magnitude of TK and LL but also the balance between the upper and lower curves is crucial [5,28]. Based on our current research findings, we feel that there is a need to focus on the upper thoracic curve and the thoracolumbar junction curve in future studies. Additionally, considering the consequences of changes in spine biomechanics after the correction of scoliosis and cervical kyphosis is crucial.

This study had two major clinical implications: First, in thoracic AIS, postoperative cervical alignment should achieve an adequate TK and promote correction of the coronal plane curve. Second, selective corrective surgery in lumbar AIS can improve postoperative cervical alignment. Finally, our study results suggest that the fixation range in AIS treatment should be reconsidered based not only on coronal plane assessment but also on sagittal plane evaluation. This approach may provide a more comprehensive and effective treatment strategy for patients with AIS, potentially improving surgical outcomes and overall spinal health.

This study had several limitations. First, it was retrospective in nature and had a small sample size. Second, it only included patients who underwent selective corrective surgery for thoracic or lumbar curves. Further, the range of fixed vertebrae was not similar. A recent report has shown that cervical sagittal plane alignment is affected by changes in TK after posterior corrective surgery in patients with upper instrumented vertebrae at the T9 level or higher [23]. Nevertheless, this study first compared postoperative cervical alignment changes between patients with Lenke type 1A curves and those with Lenke type 5C curves. Further, it evaluated the factors associated with improvement in postoperative cervical spinal alignment. Therefore, it is still valuable.

## 5. Conclusions

In thoracic AIS, postoperative cervical alignment should achieve an adequate TK and promote correction of the coronal plane curve. Moreover, selective corrective surgery can improve postoperative cervical alignment in lumbar AIS.

## Figures and Tables

**Table 1:** Baseline characteristics of patients with AIS.

Variables	Patients with AIS (*n* = 66)		
Lenke Classification	Lenke Type 1A Group (*n* = 30)	Lenke Type 5C Group (*n* = 36)	*p* Values
Age (years)	16.3 ± 2.5	16.0 ± 2.7	NS
Female/male patients (*n*)	27/3	35/1	NS
Risser grade (*n*)			NS
1	1	1	
2	1	2	
3	0	4	
4	14	13	
5	14	16	
Height (cm)	157.6 ± 3.8	158.1 ± 5.1	NS
Weight (kg)	47.4 ± 4.4	48.1 ± 5.1	NS
BMI (kg/m^2^)	19.1 ± 1.7	18.8 ± 1.9	NS
Coronal parameters			
MT Cobb angles (°)	46.4 ± 8.1		
TL/L Cobb angles (°)		39.1 ± 8.5	
Number of fixed vertebrae	5.17 ± 2.2	7.71 ± 1.1	*p* < 0.05
UIV, *n* (%)			
T4	2		
T5	19		
T6	9		
T7	1		
T8			
T9		4	
T10		8	
T11		17	
T12		7	
LIV, *n* (%)			
T11	4		
T12	11		
L1	13		
L2	2	2	
L3		32	
L4		2	

*n*, number of patients; NS, No significant difference; AIS, Adolescent idiopathic scoliosis; BMI, body mass index; MT, main thoracic curve; TL/L, thoracolumbar/lumbar curve; UIV, upper instrumented vertebra; LIV, lowest instrumented vertebra.

**Table 2:** A comparison of the alignment between the Lenke 1A group and the Lenke 5C group 1 year postoperatively.

Variable	Lenke Type 1A	Lenke Type 5C	*p* Value
C2–7 angle, °	−1.99 ± 1.43	3.88 ± 2.24	*p* < 0.005 *
C2–7 SVA, mm	21.1 ± 1.71	20.9 ± 1.58	*p* < 0.005 *
TK, °	21.2 ± 1.05	25.4 ± 1.77	*p* < 0.005 *
T1 slop, °	17.9 ± 1.37	20.7 ± 1.06	*p* < 0.005 *
LL, °	43.3 ± 1.64	43.6 ± 1.36	NS

The interval and ratio values are presented as the mean ± standard deviation. * *p* < 0.05. SVA, sagittal vertical axis; LL, lumbar lordosis; TK, thoracic kyphosis. NS, No significant difference.

**Table 3:** Correlation between C2–C7 angle and other radiographic parameters of Lenke Type 1A group.

Lenke Type 1A C2–C7 Angles (°)		Preoperatively	At 1 Year Postoperatively
C2–C7 SVA (mm) Preoperatively	r		
	*p* value	0.065	0.215
At 1 year postoperatively	r		
	*p* value	0.502	0.588
T1 slope (°) Preoperatively	r	0.367	0.427
	*p* value	0.046 *	0.023 *
At 1 year postoperatively	r	0.374	0.454
	*p* value	0.048 *	0.015 *
TK (°) Preoperatively	r	0.301	0.381
	*p* value	0.048 *	0.041 *
At 1 year postoperatively	*r*		0.424
	*p* value	0.677	0.021 *
LL (°) Preoperatively	r		
	*p* value	0.413	0.242
At 1 year postoperatively	r		
	*p* value	0.622	0.3929

SVA, sagittal vertical axis; LL, lumbar lordosis; TK, thoracic kyphosis. * *p* < 0.05.

**Table 4:** Correlation between C2–C7 angle and other radiographic parameters of Lenke Type 5C group.

Lenke Type 5C C2–C7 Angles (°)		Preoperatively	At 1 Year Postoperatively
C2–C7 SVA (mm) Preoperatively	r		
	*p* value	0.359	0.767
At 1 year postoperatively	r		
	*p* value	0.734	0.856
T1 slope (°) Preoperatively	r	0.599	0.564
	*p* value	0.0001 *	0.0004 *
At 1 year postoperatively	r		
	*p* value	0.215	0.268
TK (°) Preoperatively	r		
	*p* value	0.879	0.841
At 1 year postoperatively	*r*		
	*p* value	0.709	0.951
LL (°) Preoperatively	r		
	*p* value	0.421	0.223
At 1 year postoperatively	r		
	*p* value	0.526	0.268

SVA, sagittal vertical axis; LL, lumbar lordosis; TK, thoracic kyphosis. * *p* < 0.05.

## Data Availability

The data presented in this study are available on request from the corresponding author.

## Abbreviations

AIS	adolescent idiopathic scoliosis
LL	lumbar lordosis
PSF	posterior spinal fusion
SVA	sagittal vertical axis
TK	thoracic kyphosis
TL	thoracolumbar
